# The impact of Lego® Therapy on cognitive skills in Autism Spectrum Disorders: a brief discussion

**DOI:** 10.3934/Neuroscience.2023016

**Published:** 2023-06-30

**Authors:** Nicoletta Vegni, Caterina D'Ardia, Gloria Di Filippo, Francesco Maria Melchiori

**Affiliations:** Department of Psychology, Niccolò Cusano University, Rome, Italy

**Keywords:** ASD, Lego®, Therapy, cognitive skills, implicit learning, social skills, executive functions

## Abstract

Over the years, several interventions have been implemented, including Lego® Therapy, with the aim of supporting and implementing social and communication skills impairments in Autism Spectrum Disorders (ASD). Although recent studies have shown that the ability to learn implicitly is preserved in ASDs, no study related to Lego® Therapy has analyzed whether and how this training can also affect aspects not directly treated. In this study, we report a first attempt of assessment of Lego® Therapy's effect on the specific area of cognitive skills in an ASD child. Over a period of 12 months, a child with ASD had weekly meetings with an expert operator of Lego® aiming to improve the child's ability to communicate, reduce impulsiveness and hyper verbalism, and encourage pro-social behavior. The intervention resulted in positive outcomes that were assessed after 12 months.

## 1. Introduction

Autism spectrum disorders (ASD) are neurodevelopmental disorders characterized by persistent deficits in communication, social interaction, repetitive interests, and behaviors [1] (APA, 2013). DSM 5 [1] emphasizes the central role of developmental difficulties, but also the use of skills such as social-emotional reciprocity, non-verbal communication behaviors and the development, management and understanding of relationships.

Particularly, there are frequent difficulties in initiating interactions with peers, in sharing play activities, in using communication to share moods and in appropriately managing complex social situations that involve taking others' points of view into account [2],[3].

Several studies [4],[5] have highlighted how children with ASD, compared to neurotypical peers, have less effective interactive exchanges and, conversely, spend more time in non-social play activities. In addition, there are difficulties in using social opportunities in different contexts and reduced motivation to learn these skills [6]. This has a significant impact on the processes of testing, verifying, and learning useful social behaviors and strategies in different contexts of daily life [7]. The lack of appropriate social skills, especially during the school period, inhibits the chance to develop effective and lasting relationships, increasing the risk of isolation and reduced exchanges even in inclusive environments [4],[8]. Symptoms related to restricted behavior and interests (criterion B of DSM 5) should be differently treated. The DSM 5 [1] points out that in addition to stereotyped movements, rigidity in change and the need for sameness, the occurrence of circumscribed or perseverative interests should also be considered in this group. These patterns of behavior are often the basis for the development of abilities that can positively affect the development and evolution of ASD children. Clinical interpretative models suggest that the basis of ASD symptoms would be a delay in the development, or atypical use, of Theory of Mind (ToM) [9]–[12], Central Coherence (CCT) [13],[14] and Executive Functions (EF) skills [15],[16].

Deficits in Theory of Mind (ToM), Central Coherence (CCT), and Executive Functions (EF) not only result in challenges in social communication and interaction but also play a crucial role in repetitive interests and behaviors. Simultaneously, these weaknesses are influential in the development of visual-perceptual and visual-constructive abilities as well as intense interests in specific subjects. Paradoxically, these deficits, particularly in CCT, have been associated with high systematization skills and a propensity to think in pictures rather than words, as indicated by research [17]–[20] During the last decades, most treatment models have focused on deficits/impairments in reciprocal communication and social interaction as crucial for children with ASD. Lego® based therapy for children with ASD and related disorders stems from observations and research of LeGoff et al. [21] and is based on two assumptions: firstly, most ASD children are very skilled and interested in using Lego® and, at the same time, working with Lego® allows ASD children a common result of the building by cooperating with others. Intervention with Lego® uses restricted interest, also linked to good visual-perceptual and constructive skills, as a tool to create social exchange situations, turn-taking activities, sharing interests, learning, and using social rules [21]. Thus, it exploits CCT deficits and good systemizing skills to enhance EF and to mitigate ToM deficits. Lego® therapy uses the ability of ASD children as a motivating variable for learning and behavioral change [22]. The choice of Lego® as a tool for social development therapy is in part related to Attwood's concept of “constructive application” and the proven usefulness of using the child's interests to motivate both learning and change [23]. Many interventions for the development of social skills are affected by the choice of materials, the environment and the people involved and increase the possibility that learning will be too mechanical and hardly generalizable to external environments. The same should be applied to all interventions with a significant use of reward mechanisms [21]. Studies [24],[25] have also underlined how therapies oriented to social development are more effective when the child is confronted with one or more peers and this is linked to the value of using an effective model to learn, experiment and verify what has been acquired in a more natural system.

Indirectly, the task involves the support, or expansion, of motor praxis, visual-motor and self-control skills, management, and compliance with rules. There are a number of studies [26]–[28] that have found improvements in social development both in children with other interventions and those with no intervention. This can be partially explained by what is called implicit learning, meaning the ability to learn without being aware of it [29]. A recent review [30] has analyzed 19 articles on Lego® therapy and its application in the treatment of individuals with ASD. The review highlights the limited number of papers found (19 including only 2 single cases) and the reported results in terms of analyzed variables. All the papers focus on social, communication and interaction skills, consistent with the recommendations of Lego® therapy. Recent studies have shown how implicit learning is independent of intelligence [31]) and that this ability to learn without awareness of the learning process is not impaired in ASD [32]. It is therefore possible to hypothesize that interventions aiming at supporting lacking skills in ASDs may implicitly lead to other abilities being supported. It is therefore possible that Lego therapy could enhance cognitive skills in individuals with Autism Spectrum Disorder (ASD) through various mechanisms. Problem-solving and Planning: Lego therapy involves engaging in structured building tasks that require problem-solving skills. Individuals with ASD are encouraged to plan, organize, and execute their ideas to construct models using Lego bricks. This process enhances their cognitive abilities related to logical thinking, spatial reasoning, and sequential planning [21],[28]. Visual-Perceptual and Constructive Abilities: Lego therapy capitalizes on the visual-perceptual strengths often seen in individuals with ASD. The act of manipulating Lego bricks to create models promotes the development and refinement of visual-spatial skills. This includes recognizing patterns, understanding spatial relationships, and enhancing fine motor coordination [21],[28]. Executive Functions: Lego therapy provides opportunities to practice and improve EF, such as attention, working memory, and self-regulation. Participants need to focus on the task at hand, remember instructions and adapt their actions accordingly. These cognitive processes are essential for the successful completion of the building tasks and can have positive carry-over effects in other areas of life [21],[22]. Collaboration and Social Interaction: Lego therapy is typically conducted in small groups, requiring participants to work collaboratively towards a common goal. This cooperative play fosters social interaction, turn-taking, communication, and negotiation skills. Engaging in shared problem-solving tasks also promotes perspective-taking and understanding others' points of view, which are crucial components of social cognition [21],[22].

Generalization of Skills: The cognitive skills developed through Lego therapy have the potential to generalize beyond the therapy sessions. As individuals with ASD practice problem-solving, planning, and social interaction within the Lego context, they can transfer these skills to other real-life situations. This transferability supports the development of flexible thinking and adaptive behaviors in various contexts [21],[22]. Overall, Lego therapy harnesses the inherent appeal of Lego bricks for individuals with ASD and leverages their cognitive strengths to enhance problem-solving, visual-perceptual abilities, executive functions, and social interaction. By providing a structured and engaging environment, Lego therapy offers a unique approach to supporting and promoting cognitive skill development in individuals with ASD. The aim of this study is therefore to verify whether a Lego® based therapy, besides supporting social and relational skills, has any impact in terms of improving cognitive skills.

## 2. Materials and methods

Lego Therapy took place at the Clinical Center of the Niccolò Cusano University with a child aged 11 years and 8 months, whom we shall refer to as Vincent. V. has undergone several evaluations since kindergarten for language difficulties, reported by the teachers, and for difficulties with attention, and later, in learning. Since the early years of kindergarten, he has shown difficult relationships with peers, progressively increasing during the school cycle. During the preschool period, he underwent an initial speech therapy assessment, with a negative result. In the first year of primary school, a new assessment was carried out, showing difficulties in attention and text comprehension, for which he underwent rehabilitative therapy.

When the child entered the first year of secondary school, a new assessment was carried out, which led to a diagnosis of high-functioning ASD in association with a motor coordination disorder, mixed learning disorder, and anxious-depressive traits. Parents report a strong interest in dinosaurs, animals, and activities with Lego® since childhood. In the pre-training stage, cognitive functioning was assessed with the Wechsler Intelligence Scale for Children (WISC-IV) fourth Italian version [33], his neuropsychological profile was assessed with the Developmental Test of Visual-Motor Integration (VMI) Italian version and the Tower of London test (ToL) Italian version [34]. Finally, the ABAS II [35] was administered to parents to assess Vincent's daily living skills and level of self-reliance as reported by parents.

The preliminary assessment showed the values reported in Table 1 Vincent's cognitive profile was uneven with areas of high functioning in the Visual Spatial Index (VSI), medium-high functioning in the Verbal Comprehension Index (VCI) and borderline ranking for Working Memory Index (WMI) and Processing Speed Index (PSI) (table 1). In particular, VCI = 114, VSI = 104, WMI = 73, PSI = 71. The subscales of the WISC IV show a strong variability that makes the Overall Intelligence Quotient unreliable, so it was necessary to calculate the General Ability Index which was 106. Regarding the neuropsychological profile, in the pretraining stage, Vincent was unable to complete the task, and therefore scored in the very low range on the VMI test and ToL. The Autistic Spectrum Disorder was assessed with the ADOS 2 Test Italian version with a final comparison score corresponding to a low level of symptoms if compared with children with the same diagnosis (tot 7 - CCR4).

### 2.1. Description of intervention

The individual intervention had a frequency of one weekly meeting for twelve months, for about 90 minutes, with an expert operator of Lego® and ASD. In the first step, which lasted about 2 months, the task was focused on some specific aspects, such as the acquisition of a common language, the ability to understand what the other person was asking for and, vice versa, to make oneself understandable in the requests of the pieces and in their assembly. In addition, it was necessary to encourage the development of pro-social behavior, to reduce impulsiveness and the tendency to “do it yourself” and hyper-verbalism, not always appropriate to the context.

Following the model of Lego® Therapy V. initially built sets, exchanging roles with the operator from time to time and, in a second moment, created an original project, first illustrating it to the operator and then collaborating in its realization.

**Table 1.** Pre-training assessment scores.

	**TEST**	**Score**	**Percentiles**
**WISC**	Visual Spatial Index (VSI)	104	
	Verbal Comprehension Index (VCI)	114	
	Working Memory Index (WMI)	73	
	Processing Speed Index (PSI)	71	
	Overall Intelligence Quotient	NA	
**VMI**	Visual perception	NA	
	Motor Coordination	NA	
	Visual-Motor Integration	NA	
**ToL**		NA	
**ADOS 2**		7	
**ABAS II**	General Adaptive Composite (GAC)	57	< 1°
	Conceptual Domain	< 57	< 1°
	Social Domain	78	7°
	Practical Domain	60	< 1°

The work of the joint project required him to use what he had learned so far and to apply it consistently. At the same time, he had to discuss the realization of his own ideas with the operator. The choice fell on the creation of a city with parts of the set, built in the first stage, and new parts created specifically for the project. V. took an active part in all the steps of the common project and showed a greater ability to listen to the other and to consider the other's point of view. Depending on the moment, the operator took on the role of an external observer, to whom he had to explain what he was creating, of a helper who carried out the requests, or of an active participant who suggested ideas and solutions.

In the management of the rules written and shared at the beginning of the therapy [21] it should be noted that V. has gradually internalized and applied them spontaneously in the different sessions showing good self-regulation skills and the ability to explore his own behavior. The Lego® Therapy sessions were also used by V. as a moment of confrontation on issues not strictly related to Lego® such as films, video games but, more significantly because not solicited by the adult, on issues related to school and peers and the pursuit of functional solutions. After 12 months a new assessment was carried out, the results of which are summarized in Table 2.

#### 2.1.1. Ethics approval of research

The study has been approved by the Ethical Committee of Niccolò Cusano University as compliant to the WMA Declaration of Helsinki.

## 3. Descriptive results

As for cognitive profile and skills, in the post-training stage, Vincent achieved higher scores on all subtests (Table 2) demonstrating improvement (i.e. ranking in the high range) in several cognitive domains. Vincent's verbal comprehension index fell within the high range, whereas his visual-perceptual reasoning is in the medium-high range. His working memory index was within the medium range, with a significant increase of one standard deviation and two deviations for processing speed. Furthermore, although maintaining a certain unevenness in performance, the total IQ was measurable and stands at 114. The ADOS-2 test results confirmed a positive diagnosis of Autism Spectrum Disorder with a final score indicating a moderate level of symptoms when compared to other children with the same diagnosis (see Table 2). As for the neuropsychological profile, his scores were still confirmed relatively low, placing him in the middle range on the VMI test and in the borderline range on the ToL. Despite maintaining weaknesses in those skills, Vincent's ability to complete tasks in their entirety was signified improved.

**Table 2.** Pre and post training assessment scores.

**TEST**		**PRE-TRAINING SCORES**		**POST TRAINING SCORES**	
				Score	Percentiles
**WISC**	Visual Spatial Index (VSI)	104		126	
	Verbal Comprehension Index (VCI)	114		119	
	Working Memory Index (WMI)	73		88	
	Processing Speed Index (PSI)	71		97	
	Overall Intelligence Quotient	NA		114	
**VMI**	Visual perception	NA		104	
	Motor Coordination			98	
	Visual-Motor Integration			61	
**ToL**		NA		-1.56	
**ADOS 2**		7		8	
**ABAS II**	General Adaptive Composite (GAC)	57	<1°	59	<1°
	Conceptual Domain	<57	<1°	69	<1°
	Social Domain	78	7°	89	23°
	Practical Domain	60	<1°	46	<1°

Concerning adaptive functioning, Vincent's scores were <= 1st percentile in the general, conceptual, and practical domains. However, in the social domain, Vincent ranked in the 23^rd^ percentile, demonstrating a high level of improvement. This result supports the effectiveness of Lego® Therapy in enhancing social skills among children with ASD. Overall, these findings suggest that Vincent has made progress in several cognitive domains, despite retaining some weaknesses. The results that arise from the parental assessment expressed through the ABAS II showed a clear improvement in the Social Domain scale.

## 4. Limitations

While this single case study provides valuable insights into the participant experiences and treatment outcomes, particularly considering the clinical settings, a few relevant limitations have to be acknowledged. First of all, because single case studies focus on a single individual, it is difficult to generalize the findings to larger populations. The unique characteristics of the case may not be representative of other individuals with similar conditions, and thus the results may not be applicable to others. Secondly, the external validity is limited because the findings from a single case study may not be applicable to other settings or situations. This is particularly true if the study involves an intensive intervention or specific setting that cannot be easily replicated (e.g. when a standardized protocol has not been established, like in this case).

Another limitation is the lack of a pre- and post-intervention quantitative measure of Vincent's social skills.

Lastly, lack of control determines the potential for bias. In fact, single case methodology does not allow researchers to rule out alternative explanations for the findings. For example, there is a risk of bias in the selection of data, interpretation of results, and treatment decisions. The researcher may be influenced by their own beliefs, values, or expectations, which could impact the accuracy and objectivity of the study. The features of single case studies make it difficult to determine whether the treatment or intervention is responsible for the observed changes in the case.

## 5. Conclusions

This study aimed to investigate whether an intervention designed to facilitate the development of particular skills could have an implicit impact on other skills that were not directly targeted. Lego® Therapy was utilized in this case, which aims to promote social, relational, and communicative abilities in individuals with Autism Spectrum Disorder (ASD).

Consistent with the initial hypothesis, data collected prior to and following the treatment showed that the subject improved in the social domain, while also displaying significant advancements in terms of cognitive and EF. Prior to training, the cognitive profile was so uneven that it was impossible to provide an overall IQ evaluation. However, following training, an improvement in some subtests was observed, enabling computation and interpretation of the total IQ value. Specifically, Vincent demonstrated notable improvements on the working memory subtest by 1 standard deviation and on speed processing by 2 standard deviations. These findings appear to support the activities proposed in Lego® Therapy, which activate programming and organizational skills related to working memory and problem-solving. This is also evident in Vincent's performance on the Tower of London test, which specifically evaluates EF. Prior to training, Vincent's performance was so low that it could not be assessed. However, following training, Vincent demonstrated measurable progress and was able to complete the task, albeit with some difficulty. This brief discussion highlights the need to further investigate the link between cognitive, social and communicative skills through the application of a therapeutic program designed to increase relational skills, but which also appear to have an impact on cognitive skills.
